# A Unified Deep-Learning Model for Classifying the Cross-Country Skiing Techniques Using Wearable Gyroscope Sensors

**DOI:** 10.3390/s18113819

**Published:** 2018-11-07

**Authors:** Jihyeok Jang, Ankit Ankit, Jinhyeok Kim, Young Jae Jang, Hye Young Kim, Jin Hae Kim, Shuping Xiong

**Affiliations:** 1 Department of Industrial and Systems Engineering, Korea Advanced Institute of Science and Technology (KAIST), Daejeon 34141, Korea; natejjh2@kaist.ac.kr (J.J.); mbnmbckbs@kaist.ac.kr (J.K.); yjang@kaist.edu (Y.J.J.); 2 Department of Mechanical and Industrial Engineering, Indian Institute of Technology Roorkee, Uttarakhand 247667, India; ankit1@me.iitr.ac.in; 3 Division of Liberal Arts and Science, Korea National Sport University, Seoul 05541, Korea; hykim@knsu.ac.kr; 4 Department of Physical Education, Korea National Sport University, Seoul 05541, Korea; kimjhski@knsu.ac.kr

**Keywords:** sports analytics, deep learning, classification, inertial sensor, cross-country skiing, classical style, skating style

## Abstract

The automatic classification of cross-country (XC) skiing techniques using data from wearable sensors has the potential to provide insights for optimizing the performance of professional skiers. In this paper, we propose a unified deep learning model for classifying eight techniques used in classical and skating styles XC-skiing and optimize this model for the number of gyroscope sensors by analyzing the results for five different configurations of sensors. We collected data of four professional skiers on outdoor flat and natural courses. The model is first trained over the flat course data of two skiers and tested over the flat and natural course data of a third skier in a leave-one-out fashion, resulting in a mean accuracy of ~80% over three combinations. Secondly, the model is trained over the flat course data of three skiers and tested over flat course and natural course data of one new skier, resulting in a mean accuracy of 87.2% and 95.1% respectively, using the optimal sensor configuration (five gyroscope sensors: both hands, both feet, and the pelvis). High classification accuracy obtained using both approaches indicates that this deep learning model has the potential to be deployed for real-time classification of skiing techniques by professional skiers and coaches.

## 1. Introduction

### 1.1. Problem Definition

Cross-country (XC) skiing is a whole-body exercise endurance sport, which requires prolonged complex cyclical motions performed using skis and poles on the snow [1]. There are two main styles in XC-skiing: the classical and the skating style. The classical style can be performed both on prepared trails with pairs of parallel grooves cut into the snow or on natural undisturbed snow whereas the skating style is generally performed on firm and smooth snow surfaces. Each of the classical and skating styles have four techniques or gears. These are diagonal stride (DS), double poling (DP), push-off (P-Off), and kick-double poling (KDP) for the classical style, and V2 skate (V2), V2A skate (V2A), V1 skate (V1), and free skate (FS) for the skating style, respectively. For ease of understanding and reference, we will henceforth refer to these techniques using the abbreviations mentioned after the name of the technique.

In XC-skiing, the performance of the skiers depends on the biomechanical and the physiological aspects of the motions of the body parts and the sequence in which the skiing techniques are performed on the uphill and downhill tracks (commonly known as a natural course) and flat tracks (flat course). As the results of the skiing races can be determined by time steps as small as a few milliseconds, it becomes imperative for the professional coaches to understand both these aspects of XC-skiing to recommend an improved set of techniques for optimizing the performance of the skiers. Traditionally, these analyses have been performed using video-based systems [2,3,4,5,6] and/or force measurement systems [7,8,9]. However, the utilization of equipment in both these systems interferes with the natural movements of the body parts and the heavy cost involved limits their practical usage to only a few researchers across the world [10].

Body worn sensors, particularly the inertial sensors have recently emerged as a convenient substitute for such systems due to their small size, light weight, and low cost. Inertial sensors are sensors based on inertia and relevant measuring principles. In general, inertial sensors include gyroscopes used for measurements of the sensor’s angular velocity and accelerometers for measurements of linear acceleration. These sensors can sample at high frequencies and are easily attached to the skier’s body without interfering with the natural motion during skiing. This ease of use has made it possible to carry out experiments that require sensor data outside the controlled environment of the laboratory and provide a more realistic analysis of the task at hand. Marshland et al. [11] were the first to demonstrate this potential of body worn microsensors in the identification of XC-skiing techniques by plotting acceleration and angular velocity curves for eight athletes for both the classical and skating techniques. By visual inspection of the cyclical patterns in these plots, they concluded that all the classical and skating techniques can be clearly identified for each skier, with certain variations unique to each skier.

### 1.2. Literature Review and Proposed Work

Traditionally, studies of XC-skiing techniques have been limited to the kinematical and biomechanical analysis of various techniques [12,13]. These studies aim to determine numerous hard rules, like cycle time, poling/pushing time, recovery time [5,14,15], number of recovery motions, the sign of forearm angular velocity [16,17], correlation of the angular velocity of arms and legs [18], and figures showing identifiable cyclic patterns in the gyroscope and accelerometer data for the classification of techniques. These approaches, however, are extremely time-consuming as the derivation of the classification rules requires manual analysis of the gyroscope and accelerometer data from multiple sensors. Recently, many researchers have analyzed and classified techniques of XC-skiing using algorithms, like markov chains of multivariate distributions, and more advanced machine learning techniques. Stoggl et al. [19] utilized an accelerometer attached to the chest of professional skiing skiers to classify skating techniques. They collected data of 11 skiing skiers on a treadmill and developed a classification model based on the markov chains of multivariate distributions. Their model achieved an accuracy of 86% ± 8.9% on the test set when the training data included data from all the skiers, which rose to 90.3% ± 4.1% when separate classification models were developed for each skier. Rindal et al. [1] utilized a neural network for the classification of skating techniques by utilizing two sensors-a gyroscope on the arm for cycle identification and an accelerometer on the chest for technique classification. They achieved an accuracy of 93.9% on the test set. In both studies, the raw data was passed through a gaussian filter for the removal of ringing effects and undesirable time shifts at different frequencies. Ristner [20] implemented a Markov model and a k-nearest neighbors (KNN) algorithm for classifying XC skiing techniques using a 3D accelerometer attached on the chest of the skiers. The comparison showed that the KNN algorithm showed much lower error rates (0.19%) than the Markov model (7.22%). All these studies are impressive and show promise for the automatic and reliable classification of XC-skiing techniques using inertial sensors. However, these studies suffer from many limitations. In the study performed by [19], the data is collected in the controlled environment of the laboratory, which will be different from the actual on-field data. The neural network model used by [1] takes in data of each cycle after flattening it into a single vector. This leads to information loss as the spatial and temporal patterns in the data are lost. Both studies develop models that classify either only the classical or skating techniques and do not obtain a single model, which could be employed for both the styles. Table 1 summarizes the relevant details of the aforementioned studies.

In this paper, we propose a unified convolutional neural network (CNN) and long-short term memory (LSTM) based deep learning classification model, which can be used to classify both the classical and skating style techniques of the skiers simultaneously. The first novelty of our approach lies in using convolutional layers for merging the local interactions among the time-series data obtained from each sensor and recurrent layers (long-short term memory layers) for extracting the temporal patterns. In this way, the model is able to extract important features for the classification of various techniques automatically from the raw data, thus eliminating the need for manually designed features required by machine learning algorithms. To prove this point, we present a comparison of the results obtained from our model with a KNN model developed by manually extracting features on the same training, validation, and test datasets. We collected the flat and natural course data of four professional skiers in total and pose a working hypothesis that the generalization accuracy of the proposed deep learning model increases as the amount of training data is increased. To prove this point, the model is first trained on the flat course data of two professional skiers and tested on the flat and natural course data of a third skier in a leave-one-out fashion. Secondly, the model is trained over the flat course data of three skiers and tested on the flat and natural course data of the fourth skier. An increase in the accuracy of classification when the size of the training data is increased confirms this hypothesis.

The second novelty of our approach lies in developing a unified model, which can be used for the classification of both classical and skating techniques simultaneously. We present strong evidence in favor of using only the flat course data for training the model and using it to classify XC-skiing techniques both on flat and natural courses, thus eliminating the need for collecting natural course data for training, which is extremely difficult to procure. Finally, the comparison of accuracies among five different combinations of sensors, which establishes the sports biomechanics configuration (both hands, both feet, and the pelvis sensors) as the optimal set of sensors, provides empirical evidence to researchers to base their future studies on this optimal configuration.

## 2. Materials and Methods

### 2.1. Inertial Sensors, Synchronization and Calibration

**Figure 1:**
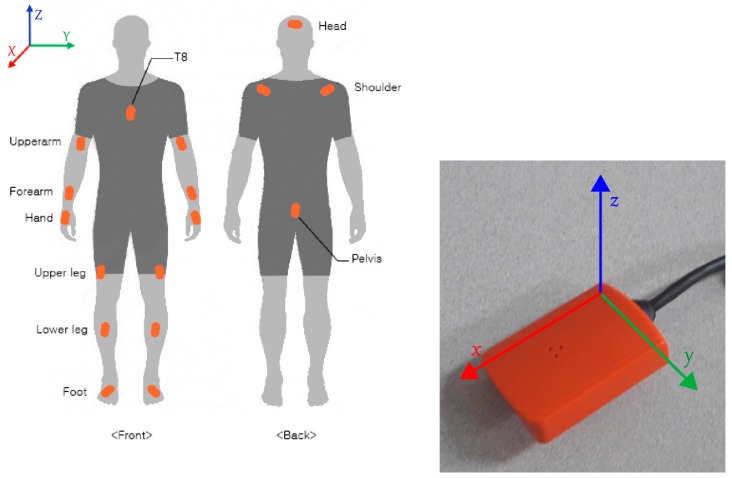
(**Left**) Front and back view of the position of 17 inertial motion trackers that are attached to the Xsens bodypack worn by the skiers. (**Right**) One Xsens inertial motion tracker depicting the local *x*, *y*, and *z* axes.

XSens MVN motion capture system (Xsens Technologies B.V., Enschede, The Netherlands) consisting of 17 body-wired inertial motion trackers (Figure 1) was used to record the participants’ kinematical data at a frequency of 240 Hz. Each motion tracker is 36 × 24.5 × 10 mm in dimension, weighs 10 g, and is mounted at a specific body location with the help of a wearable lycra suit. The wired motion trackers are connected to an on-body data hub (known as bodypack), which is responsible for synchronization and gathering data on its internal memory (Xsens MVN Technical Report, March 2018).

Calibrations were performed by the system software, *Xsens MVN Analyze*, prior to data collection, which requires the subject’s height and foot length to estimate the dimensions and proportions of the person being tracked. After subject calibration, he/she is asked to stand still in a T-pose and walk a few meters back and forth for the purpose of the sensor to segment calibration and development of a biomechanical human model. This is followed by a slight forward movement for defining the positive *X*-axis. The *Y*-axis is perpendicular to the *X*-axis in the horizontal plane while the *Z*-axis is perpendicular to the horizontal surface. Each motion tracker has 3 sensors: accelerometer, gyroscope, and magnetometer, which provide raw recorded data of linear acceleration, angular velocity, and magnetic field intensity along the *x*, *y*, and *z* axis local to each sensor, respectively.

The on-body recording function was used for data collection, which allows recording of the subject’s motions without the need for a laptop or PC by storing the motion trackers’ data on the body pack. After finishing the data recording, the body pack was connected with a laptop to import the recordings for further processing and analysis.

### 2.2. Data Acquisition

#### 2.2.1. Training and Validation Data Acquisition

For the purpose of training and validating the trained model, XC-skiing data from 3 professional skiers (Table 2) from the Korea National Sport University was collected. They performed the classical and skating XC-skiing techniques on outdoor flat and natural courses in Pyeongchang, South Korea, where the 2018 Winter Olympic Games took place. All of them were informed of the purpose of this study and they participated voluntarily in the experiment after reading the research guidelines and signing consent forms. The study was ethically approved by the Korea National Sport University Institutional Review Board (IRB Number 20170424-004).

In the training dataset, each skier performs only one technique of either the classical or skating styles on the flat course repeatedly. Each subject performs 5–6 laps of each technique on a 500 m long track. As there are 8 techniques (4 classical and 4 skating) and 3 skiers, a total of 24 such files are obtained. In the validation dataset, each skier is allowed to perform either all the 4 classical or skating techniques on either a 2.5 km long flat or natural course, similar to what he/she would perform under competitive conditions. The skiers are free to make transitions from one technique to the other; however, the skiing style remains the same. A total of 11 files are obtained in this manner (1 file could not be obtained due to unavailability of the tracks). For both the datasets, a video recording of the skiers while performing the skiing techniques is also shot. Table 3 lists the type of data collected for each subject. In order to examine whether our developed model could classify skiing techniques for skiers with different skill levels, we allowed the skiers to freely choose their own preferred skiing speeds and exercise intensities during the data collection.

Each file in the training data exclusively contains the data of one of the techniques of one of the skiing styles, and hence does not require any labelling. However, the 4 techniques of each XC-skiing style in validation data are performed in a combined way and hence have to be labelled after data collection. The ground truth labels for the validation data are developed by professional cross-country skiing players from the Korea National Sport University by simultaneously watching the recorded video from a digital camera, human model video from the *XSens MVN Analyze*, and marking the frames corresponding to each technique in the raw data files. Labelling follows a 0–9 convention for each file: 0: start/end of the recording, 1: DS, 2: P-Off, 3: KDP, 4: DP, 5: V2, 6: V2A, 7: V1, 8: FS, 9: descending). The labels have been double checked by a professional XC skiing coach at the Korea National Sport University.

#### 2.2.2. Test Data Acquisition

For the purpose of testing the generalization of the trained model, two test datasets of a new skier (skier 4) were collected. The test subject (gender: Female, age: 24 years, weight: 55 kg, height: 156 cm) is also a professional XC-skiing player from the Korea National Sport University and prior information about the purpose of the study was provided to her followed by signing of consent forms (IRB Number 20170424-004).

The 2 types of test data are as follows:

(i) Test set 1: In the first type of test data, the subject is allowed to perform only one of the techniques of one of the XC-skiing styles on the flat course. As there are 4 classical and 4 skating techniques, this test set consists of 8 files.
(ii) Test set 2: In the second type of test data, the subject performs all the skating style techniques on a natural course simultaneously. The subject is allowed to make transitions between the various skating techniques similar to what would be performed during a competition. One data file is obtained in this manner.

### 2.3. Data Selection and Preprocessing

**Figure 2:**
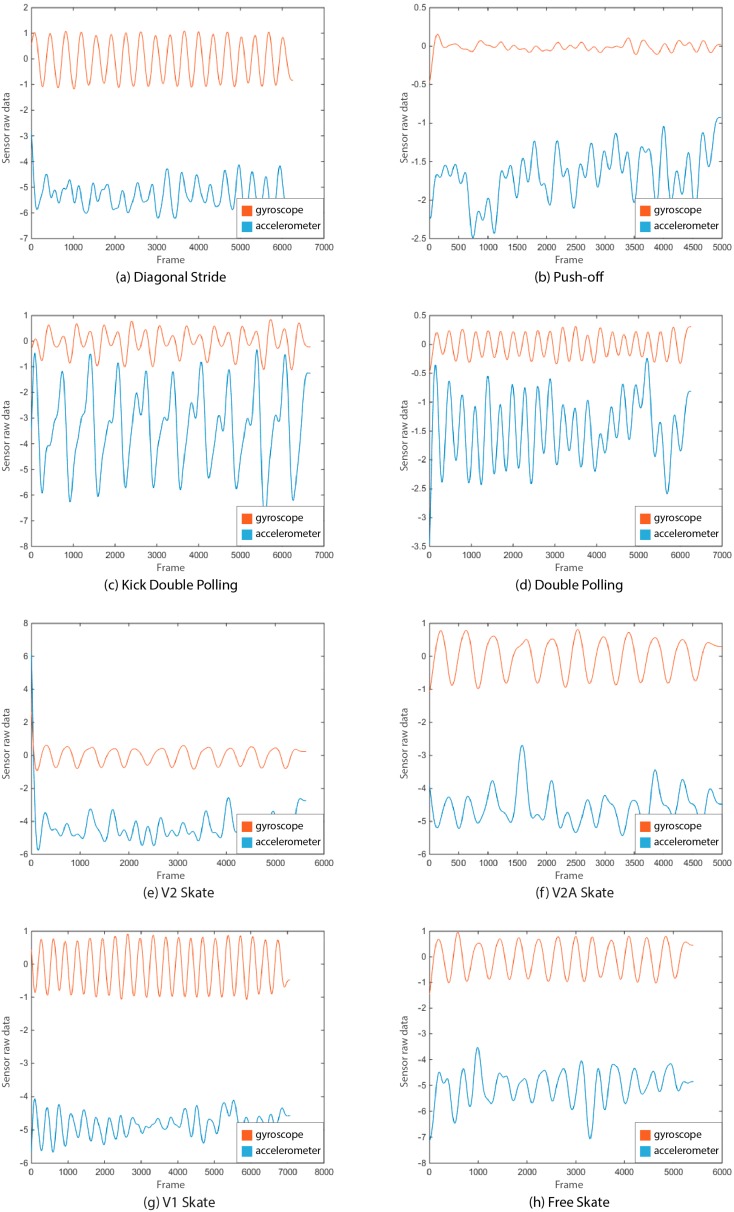
Comparison of the cyclic patterns in the *z*-axis (anteroposterior direction: normal to frontal plane) angular velocity and linear acceleration data (from a motion tracker on the flat surface of the shin bone of left leg) for the classical (**a**–**d**) and skating (**e**–**h**) XC- skiing techniques.

In this study, each training instance represents one cycle of one of the techniques of either the classical or skating styles. Thus, it becomes extremely important to select the data that can represent these cyclic patterns most clearly. Figure 2 represents the typical data patterns in linear acceleration and angular velocity data for each of the 4 classical and 4 skating techniques. These figures were plotted after filtering the raw data using a low pass butterworth filter of fourth order and a cutoff frequency of 0.007 Hz. As is clear from the figure, angular velocity data, which is obtained via the gyroscope, shows more easily identifiable cyclic patterns as compared to linear acceleration data, which comes from the accelerometer. Due to the ease of identifying the cycles and low computational cost from the less sensor data, only the gyroscope data is used in this study for developing the classification models.

#### 2.3.1. Training Dataset

Each of the 24 files in the training dataset, in which each skier performs only 1 technique of either the classical or skating style on the flat course repeatedly, has turning points at the end of a lap each time the track is traversed. During the turning points, the skier is not performing any technique and hence these points are removed from the data of each skier by manually identifying the frames corresponding to such durations using the Xsens human model videos and the recorded videos from a digital camera. This gives data files with continuous repetitions of the same technique. A low-pass butterworth filter of fourth order and a cutoff frequency 0.007 Hz is applied to smoothen the raw data, following which the *z*-axis angular velocity of the gyroscope on the left leg is used for finding the locations of peaks. The distance between two consecutive peaks in the filtered data is variable and represents 1 cycle. Each input to the CNN-LSTM model must have the same dimensions. Thus, the locations of the peaks in the filtered data is used to resample the raw data (after removing the turning points) to a fixed cycle-length of 333 time-steps (which is the mean number of time-steps for all the techniques in the training data) using an anti-aliasing finite impulse response low pass filter, which resamples the data at (333/n) × 240 Hz, where n is the number of time steps in a given cycle before resampling and 240 Hz is the original sample rate. Resampling is performed over the raw data and not over the filtered data. It is because a neural network works best with raw data from which it automatically extracts the features required for the classification task. These resampled cycles are arranged into tensors, which make it suitable for passing them to a CNN layer and later to an LSTM layer as a multivariate time series. We thus obtain a total of 24 tensors. The number of cycles of each technique of each XC-skiing style performed by each skier on the flat course is shown in Table 4.

The dimensions of each matrix in a tensor is 333 × 51 (17 gyroscopes, 3 axes each, hence 51 columns).

#### 2.3.2. Validation Dataset

In the validation dataset, each skier performs either all the 4 classical or skating techniques on either a flat course or a natural course simultaneously. Each of the 11 files in the validation dataset has starting and end points of the recording (labelled as 0), descending points (labelled as 9), and transition points (not labelled), which are classified as noise. The start/end and descending points are deleted from the data and the transition points are removed by manually identifying the frames corresponding to such time durations using the Xsens human model videos. The filtering of this data for cycle detection followed by resampling and arrangement into tensors is in accordance with what was performed for the training dataset. A total of 11 tensors are obtained in this manner and the dimensions of each matrix in a tensor is 333 × 51 (17 gyroscopes, 3 axes each, hence 51 columns). Table 5 provides further information about this dataset.

As can be observed from Table 5, different skiers have different preferences in terms of the techniques they use on a particular course. For example, skier 1 does not perform double poling (DP) on the flat course at all whereas skier 2 performs it 26 times and skier 3 performs it 46 times (highest among all classical techniques) on the same course. However, it is clear that there is a somewhat more even distribution among the usage of skating techniques as compared to classical techniques.

On the natural course, all the skiers do not use free skate (FS) and seldom use push off (P-Off) and kick double poling (KDP). Thus, it is clear that the skiers have certain preferences on the techniques they utilize on different courses (Table 5). This preference makes this dataset highly imbalanced and a perfect validation set for validating the performance of the trained deep learning model.

#### 2.3.3. Test Dataset

In test set-1, the test subject (skier 4) performs only one of the techniques of one of the XC-skiing styles repeatedly on the flat course. It has 8 files (one file for each technique of the classical and skating styles) and resembles the training data. Thus, its preprocessing is performed analogously to the training data. Similarly, the test set-2 resembles the validation data and its preprocessing is performed analogously to the validation data. Thus, 8 tensors are obtained for test set-1 and 1 for the test set-2. Table 6 contains more information about the 2 types of test sets.

### 2.4. Architecture of the Deep Network

The deep learning model developed to classify the XC-skiing techniques is motivated from Deepsense proposed by Yao et al. [22], which in the authors’ words “provides a general signal estimation and classification framework [for regression and classification problems] that accommodate a wide range of applications.” The training, validation, and testing data of our problem is arranged into 3D tensors, where each matrix corresponds to one training (or testing) example and has 333 rows and 51 columns. Each column contains data along one of the axes as recorded by one sensor and represents a time-series with 333 time steps. Thus, the classification problem is posed as a multivariate time-series sequence classification task. To capture the interactions among these time series, they are passed through convolutional layers. Deepsense first performs fourier transformation on the raw data of each sensor, passes the frequency data of each sensor through convolutional layers individually, and then combines the data of all the sensors to pass it through another convolutional layer. This approach requires a greater number of convolutional layers and selecting the number of frequencies that must be passed to the network, which introduces an element of human decision-making. We solve this problem by making 2 simple modifications to our model: (i) We pass the raw sensor data to the convolutional layers instead of the frequency data, and (ii) we convolve the raw data of all the sensors in a single step, which reduces the total number of convolutional layers required for training.

**Figure 3:**
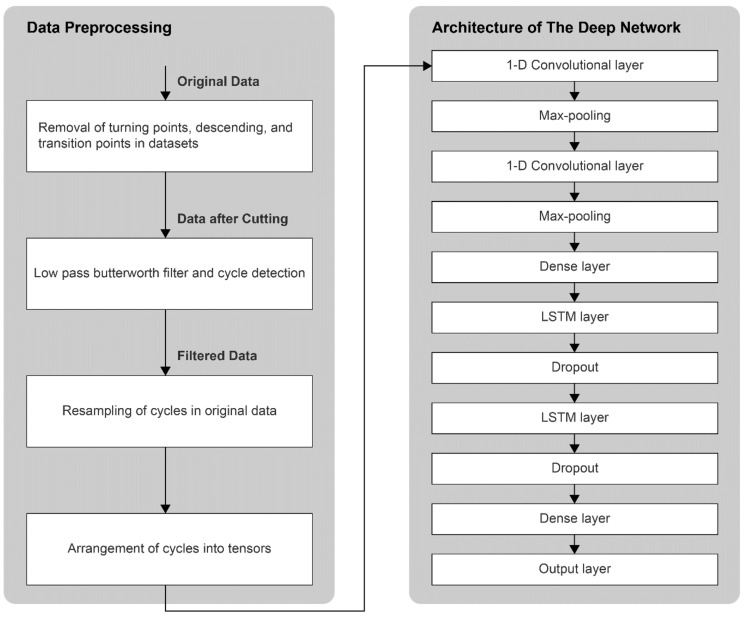
(**Left**) A schematic view of the steps involved in preprocessing of the raw data, and (**Right**) the architecture of the deep neural network.

As the skiers may perform the same skiing techniques at varying speeds and intensities, it is necessary that the deep network layers be robust to the scale of the data and be able to capture features that may be found at different time-steps in the time-series. CNNs are very powerful in extracting local spatial coherence and dependencies in the data, and the scale invariance introduced by the max-pooling layers allows them to learn hidden features regardless of the position of the feature or its scale. We pass the raw data through two 1D convolutional layers with 64 filters each and 20 and 10 kernels, respectively, followed by a max-pooling layer with a pool-size of 4. This convolved data is then passed through 2 long-short term memory (LSTM) layers, with 300 and 200 units, respectively, to capture long-term temporal dependencies in the time series. As LSTMs are highly prone to overfitting, a dropout layer with a dropout probability of 0.2 is added after each LSTM layer. The network is trained over 12 epochs with a batch size of 40. Various steps involved in data preprocessing and the architecture of the deep network are summarized in Figure 3.

## 3. Results

We now present results for the training, validation, and test sets obtained by training the proposed CNN-LSTM based deep learning model. To compare the results with a traditional machine learning algorithm, we also trained a k-nearest neighbor (KNN) classifier by extracting manually designed features from the training data. Five different combinations of sensors are used for training the model as shown in Table 7. We will compute and compare the results for each of the sensor configurations and come up with the best subset of the 17 sensors, which should be used for the analysis of XC-skiing techniques in future studies.

### 3.1. Training and Validation Set Results Using Deep Learning

The deep learning model, trained on the training data of the three skiers according to the architecture described in Section 2.4, resulted in a training dataset accuracy of at least 97.8% for the whole body, upper body, lower body, and sports biomechanics sensors configuration, and 79.4% when only the pelvis sensor is used (Table 8). Table 9 provides the confusion matrix for the training dataset for the sports biomechanics configuration.

As is clear from Table 9, the model is able to classify all the techniques almost perfectly except the classical push-off and double poling techniques. Twenty-eight push-off techniques have been wrongly classified as double poling techniques and nine double poling techniques as push-off techniques.

The validation dataset accuracies for the five different configurations of sensors are shown in Table 10.

The mean classification accuracy with the pelvis, the upper, and the lower body sensors is 64%, 80%, and 70% respectively, which increases to approximately 87% for the 17 sensors (the whole body) and the sports biomechanics configuration. As the accuracies while using all the 17 sensors and the five sensors in sports biomechanics configuration are the same, the sports biomechanics configuration of sensors is the optimal set due to much smaller number of sensors. The confusion matrices for the validation sets for skier 3 for the sports biomechanics configuration are shown in Table 11, Table 12, Table 13 and Table 14. The confusion matrices for the validation sets of skier 1 and skier 2 can be found in Appendix A.1 and Appendix A.2.

It is interesting to note that although the model has been trained on classical and skating styles data simultaneously, it has almost perfectly learnt to differentiate between these two styles. In Table 12, three V2 techniques have been incorrectly classified as V2A, and 23 out of the 28 V2A techniques have been incorrectly classified as V1. In Table 13, five push-off techniques have been incorrectly classified as DP and 6 KDP techniques as push-off. These are certain areas of misclassification errors, which the model is not robust to. However, despite these small misclassification errors, the mean classification accuracy achieved for skier 3 is approximately 90%, which is a very high value considering that our model is trained only on the flat course data and has never observed natural course data.

### 3.2. Leave-One-Out Testing Results

To assess the generalization accuracy of the model, we perform a leave-one-out type of testing in which the flat course data of two out of the initial three skiers is used for training and the flat and natural course data of the remaining third skier is used for testing. As the third skier can be chosen in ^3^C_1_ = 3 ways, we have a total of three combinations of training and test sets. For example, combination 1 includes subject 2 and subject 3’s flat course data as the training set, and subject 1’s flat and natural course data as the test set. We present the results of leave-one-out testing for both the proposed deep learning model as well as a traditional k-nearest neighbors (KNN) machine learning algorithm. For the KNN algorithm, the feature vector corresponding to a cycle in the training data consists of the pairwise correlation values between the time-series represented by each axis of each sensor in a cycle. For example, while using the whole-body configuration of sensors (17 sensors), there are a total of 51 time series in each cycle, which correspond to a feature vector of length 1276 (=^51^C_2_ + 1). Table 15 presents the results of the proposed deep learning model and Table 16 for the KNN machine learning model when a leave-one-out type of testing is performed using the sports biomechanics configuration of sensors. The results for the other four configurations of sensors are available in the Appendix A.3.

The overall mean accuracy for the leave-one-out type of testing for the three skiers using k-nearest neighbors’ algorithm is ~65%, which increases to ~80% when the proposed deep learning model is used. Also, the mean accuracy values for each skier is higher in the case of the deep learning model as compared to the KNN model.

### 3.3. Test Set Result Using Deep Learning

To further assess the generalization performance of the model, it is trained on the flat course datasets of the first three subjects and tested on the two test datasets obtained from subject 4. The classification accuracies for test set-1 and test set-2 for all five sensor configurations are as shown in Table 17. Again, the sports biomechanics configuration with five sensors has maximum accuracy for both the test sets, reaffirming the aforementioned proposition that this configuration is the optimal set of sensors.

Table 18 and Table 19 show the confusion matrices for test set-1 and test set-2 for the sports biomechanics configuration, respectively. In test set-1, all the techniques except the classical push-off (P-Off) and double poling (DP) have been classified almost perfectly. One hundred and seventy-two (out of total 241) push-off techniques have been incorrectly classified as double poling and 74 (out of total 295) double poling as push-off, leading to a low classification accuracy for the push-off and the double poling. These are the same two techniques that were confused by the model in the training set, and hence some misclassification in the test set was also expected. For test set-2, a very high overall accuracy of 95.1% is obtained for the sports biomechanics configuration of sensors. Thus, we achieve an overall mean accuracy of 91.15% on the test set of skier 4.

It should be emphasized that the overall mean accuracy when a leave-one-out type of testing is performed over the first three skiers is ~80%, which increases to ~91.1% when testing is performed over skier 4 using the same deep learning model. This is due to the fact that the deep learning model tested over skier 4 has been trained over the data of three skiers whereas the same model when tested over each of the first three skiers has been trained over the data of two skiers in a leave-one-out fashion. Thus, the generalization accuracy of the proposed model increases as the size of the training dataset is increased. These results provide strong evidence in favor of our hypothesis that the accuracy of the deep learning model increases as the training datasets become larger.

### 3.4. Validation and Test Set Results Using K-Nearest Neighbors Algorithm

To compare the results of the deep learning model with a traditional machine learning algorithm, we trained a k-nearest neighbors classifier on the training data of three subjects and tested it on the fourth subject. The feature vector corresponding to a cycle in the training data consists of the pairwise correlation values between the time-series represented by each axis of each sensor in a cycle.

Table 20 represents the validation set and Table 21 represents the test set-1 and test set-2 accuracies for all five configurations of the sensors.

## 4. Discussion

**Figure 4:**
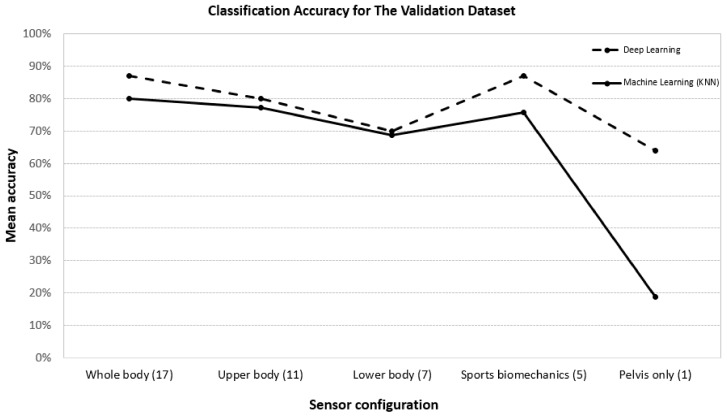
Comparison between the machine learning (KNN) and deep learning methods on classification accuracies for the validation dataset for the five different sensor configurations.

We developed a unified CNN-LSTM based deep learning model for classifying both the classical and skating style techniques simultaneously using the gyroscope data. Even though our model was trained only on the outdoor flat course data, it achieved an accuracy of 87.2% and 95.1% on the flat and natural course test sets, respectively, leading to an overall mean accuracy of 91.15%, using the optimal gyroscope sensor configuration (five sensors: both hands, both feet, and the pelvis). This presents strong evidence in favor of using only the flat course data for training the model and using it to classify XC-skiing techniques both on flat and natural courses, thus eliminating the need for collecting natural course data for training, which is extremely difficult to procure. To the best of our knowledge, we are the first ones to propose a unified deep learning model for classifying classical and skating techniques simultaneously with high accuracy. A KNN model with manually designed features for the skiing technique classification on the same datasets was further used as a benchmark for evaluating the performance of our unified deep learning model. The KNN algorithm was chosen since it is preferable due to less error rates in classifying the classical style and skating style simultaneously when compared with a Markov model according to an earlier study [20]. The comparison between the accuracies obtained from these two approaches for the validation dataset (Figure 4) and two test sets (Table 22) clearly showed that deep learning is more effective and has higher classification accuracy than the KNN. This result is in line with the findings from a recent study[23], which used a 3D accelerometer to classify only two free skating style techniques (gear 2, gear 3) and reported that the deep learning had the highest accuracy among all investigated classification models.

Even though the developed deep learning model achieved high overall classification accuracy for eight skiing techniques simultaneously, in-depth analysis of the confusion matrices showed that most incorrect classifications occurred for classical push-off and double poling techniques. The classical push-off and double poling techniques have identical motions of upper body and pelvis, the only differences between these two techniques are that a classical push-off begins with a slight jump for the propulsive force and the body movements are faster and exaggerated as compared to double poling. Such exceedingly similar physiological and biological characteristics are the cause for the confusion of the model and lead to misclassifications. In addition, some misclassifications occurred for V2A, which is a typical technique used in level terrain up to moderate uphill inclines or during transitions between V2 and V1. In V2A skate, the timing sequence for pole push is the same as V2 skate, but it employs one double pole with every second skate, which is different from one double pole with every skate in V2 skate**.** V1 skate is an uphill technique, which employs an asymmetrical poling with every second skate [3,24]. Transitions between similar techniques, V2A and V2, V2A and V1 lead to high classifications errors on V2A. This finding is consistent with the result from a previous study [19].

In order to provide empirical evidence to researchers to base their future studies on the optimal sensor configuration for analysis of XC-skiing techniques, we compared classification accuracies among five different combinations of sensors on the training, validation, and test datasets. The five combinations include whole body with 17 sensors, upper body with 11 sensors, lower body with 7 sensors, sports biomechanics configuration with 5 sensors, and the pelvis configuration with 1 sensor only. Collective results (Table 10, Table 20 and Table 22) show that the sports biomechanics configuration (both hands, both feet, and the pelvis sensors) can achieve a very similar accuracy as the whole body with 17 sensors. The classification accuracy from the sport biomechanics configuration is much higher than the accuracies from the pelvis, the upper, and the lower body sensors. A low classification accuracy for the pelvis sensor indicates that this sensor alone is not sufficient to capture the complex motions of all the body segments during the XC-skiing. Moderate, but not high, classification accuracies for the upper and lower body configuration of sensors are not surprising because in both the configurations, the data of only half of the body segments is available for training the model. In the sports biomechanics configuration, only five sensors, those on the hands, on the feet, and the pelvis, are used. Out of these five body segments, four body segments, both hands and feet, are at the extremes of the body where the motions of the segments are most exaggerated and vigorous, and the pelvis is close to the centre of the mass of the body, which represents an overall motion of the body segments. As the results while using all 17 sensors and five sensors in sports biomechanics configuration are very close to each other, we infer that the other 12 sensors are almost inconsequential and provide no additional information. Thus, the sports biomechanics configuration of sensors is the optimal set and future studies of XC-skiing classification can be based on the data obtained from this set with strong experimental proof.

Several previous studies have attempted to classify XC-skiing techniques by numerous hard rules or machine learning algorithms. Seeberg et al. [18] classified the classical XC-skiing techniques by deriving hard rules based on the data of 11 skiers and achieved an overall sensitivity of 99~100%. They, however, classified only the diagonal stride, double poling, and kick double poling techniques while leaving out push-off from the classification. Among the classical XC-skiing techniques, push-off and double poling are the only two techniques that are substantially misclassified by our algorithm, as is evident from the test set-1 confusion matrix in Table 18. One hundred and seventy-two (out of total 241) push-off techniques have been incorrectly classified as double poling and 74 (out of total 295) double poling as push-off. Moreover, they utilized six IMUs for classification of XC-skiing techniques and a total of 18 sensors were used, since each IMU contains one accelerometer, one gyroscope, and one magnetometer, whereas our model performs classification only with five gyroscope sensors. In addition, our model development does not require expert domain knowledge and a tedious process to derive the hard rules for classification. Rindal et al. [1] classified classical XC-skiing techniques by utilising two sensors, one accelerometer and one gyroscope, on the data of 10 participants and achieved an overall accuracy of 93.9% ± 3%. We achieved an overall mean accuracy of 91.1% by utilising data of four subjects and five gyroscope sensors. Our results rivals the results of [1] in terms of accuracy, but at an additional cost of three extra sensors. However, the data in [1] is a combination of data obtained from outdoor tracks and that obtained on a treadmill in the controlled environment of the laboratory whereas our data is obtained only from natural outdoor tracks. Additionally, they classified only the classical XC-skiing techniques whereas our model classifies both the classical as well as the skating techniques simultaneously. At the same time, our model shows considerable improvement in classification accuracy when the size of the training data is increased. Stoggl et al. [19] classified skating techniques by utilising a single accelerometer on the data of 11 skiers obtained on a treadmill in the controlled environment of the laboratory, and achieved an accuracy of 86% ± 9% on the test set. As the accuracy achieved by our model is higher and our model has additional advantages in terms of performing classification of both classical and skating techniques simultaneously, we conclude that our model has higher potential of being deployed as a real time classification model for XC-skiing techniques.

Despite the inherent advantages in terms of automatic selection of features, high accuracy, and simultaneous classification of classical and skating XC-skiing techniques, this study suffers from certain limitations. First, due to practical constraints, such as the unavailability of the skiing tracks and tight training schedule of professional skiers, we only obtained the experimental data from a relatively small sample size (four professional skiers) for training, validating, and testing our models, further study should be carried out with a larger sample size for the verification of these results. Second, although the CNN-LSTM network promises high accuracy, the model is slow to train as compared to a model developed using a traditional machine learning algorithm due to the large size of training data that is fed to it. Traditional machine learning approaches rely on manually designed features, compact the raw data into a small number of features after pre-processing, and are much faster to train. Thus, there is a compromise between the time spent in data pre-processing in the case of traditional algorithms and training a deep learning model. However, by utilising computer systems with good software configurations, the deep learning model can be trained in a reasonable time to remain suitable for real time deployment. In this study, we assumed turning points in flat course data and descending and transition points in natural course data as noise and removed them manually by finding frames corresponding to them. These points, however, can be treated as dummy techniques and passed to the model, the study and analysis of which should be taken up as future research work. Last, but not the least, we utilized only the angular velocity due to clear cyclic patterns and for achieving a higher test time efficiency. The development of classification models using linear acceleration and magnetic fields is left as a future research work.

## 5. Conclusions

We utilized a novel CNN-LSTM based deep learning approach to develop a unified model for the classification of eight techniques used in classical and skating styles for XC-skiing. Overall, we achieved an accuracy of 87.2% and 95.1% on the flat and natural course test sets using the optimal sensor configuration (five gyroscope sensors: both hands, both feet, and the pelvis). High classification accuracy on both the test sets indicates that this deep learning based approach is very promising for automatic identification and classification of different XC-skiing techniques. The essence of our approach lies in eliminating the need of manually designed features required for traditional machine learning approaches and substituting the video-based and force measurement systems for classification of the XC-skiing techniques. Our model has the potential to be trained in the wild and as data of more skiers is made available, the fine tuning of the parameters will improve the accuracy as well as the scope of generalization continuously. This increases the practical value of our model and makes it suitable for real-time deployment by sports professionals. We optimized for the number of sensors and obtained the sports biomechanics configuration with five sensors as the optimal set, providing empirical evidence to researchers to base their future studies on this optimal configuration.

## Figures and Tables

**Table 1:** Previous studies on XC-skiing technique classification with wearable sensors.

Related Work	Number of Sensors/Locations	Subjects	Number of Classes (XC-Skiing Style)	Used Classification Method	Classification Accuracy	Data Acquisition Details
[18]	6 inertial measurement units (IMUs)/upper back, lower back, left and right arm, left and right ankle; 1 sensor unit/chest (including 1 accelerometer, 1 gyroscope, 1 skin temperature sensor, 1 heart rate sensor)	11 (10 males, 1 female)	3 (classical style)	An algorithm based on the correlations of angle values of arms and legs	Sensitivity: 99~100%	Data collected in different types of tracks and snow conditions
[19]	1 accelerometer/chest	11 (7 males, 4 females)	5 (skating style)	Machine Learning Model (Markov Chain of multivariate Gaussian distribution)	86% ± 8.9% for collective data	Data collected on treadmill using roller skies (different speeds and inclines)
[20]	1 accelerometer/chest	3 skiers for the test set	Both classical style and skating style	Machine Learning Models (a Markov model and a KNN model)	The error rates for tests: 7.22% for the Markov model; 0.19% for KNN model	The test set for the cross-validation consists of 30 cycles from each gear from three different skiers in classical style and from three different skiers in skating style
[1]	2 inertial sensors: 1 accelerometer/chest; 1 gyroscope/arm	10 (9 males, 1 female)	8 (classical style)	Neural Networks	93.9% ± 3% for the test data	Data collected on treadmill and real competition course on snow
[21]	1 accelerometer/chest	No details	2 (skating style)	Neural Networks	CNN error rate: 2.4% LSTM error rate: 1.6%	No detailed information

**Table 2:** General characteristics of the three professional skiers that participated in the study.

	Attribute	Gender	Age (in Years)	Weight (in kg)	Height (in cm)
Skier					
1		Female	24	51	163
2		Female	22	51	162
3		Male	23	69	176

**Table 3:** Training and validation data collected for three professional skiers characterized by the type of course (flat/natural) and the number and type of skiing techniques (classical/skating) that the subject is allowed to perform simultaneously.

Skier	Training Dataset	Validation Dataset			
	Flat Course; 1 Technique of Skating or Classical Style Repeatedly	Flat Course		Natural Course	
		Classical Style (DS, P-Off, KDP, DP)	Skating Style (V2, V2A, V1, FS)	Classical Style (DS, P-Off, KDP, DP)	Skating Style (V2, V2A, V1, FS)
Skier 1	√	√	√	X	√
Skier 2	√	√	√	√	√
Skier 3	√	√	√	√	√

X: The classical style data on the natural course for skier 1 is not available.

**Table 4:** Number of cycles of the classical (DS, P-Off, KDP, DP) and skating (V2, V2A, V1, FS) techniques performed by each skier in the training dataset.

	Technique	Classical Style				Skating Style				Sum
Skier		DS	P-Off	KDP	DP	V2	V2A	V1	FS	
Skier 1		153	123	107	128	85	104	157	103	960
Skier 2		103	83	94	68	38	56	65	61	568
Skier 3		83	64	64	78	48	59	86	57	539
Sum		339	270	265	274	171	219	308	221	2067

**Table 5:** Number of cycles of each technique for the classical and skating style performed by each skier in the validation dataset on the flat and natural course.

Skiing Course & Technique	Flat Course		Natural Course	
	Classical Style (DS, P-Off, KDP, DP)	Skating Style (V2, V2A, V1, FS)	Classical Style (DS, P-Off, KDP, DP)	Skating Style (V2, V2A, V1, FS)
Skier 1	33, 13, 39, 0	44, 60, 7, 21	X	60, 31, 197, 0
Skier 2	49, 0, 54, 26	13, 24, 15, 10	190, 0, 7, 63	98, 34, 602, 0
Skier 3	16, 27, 34, 46	23, 32, 19, 19	145, 2, 5, 85	89, 30, 44, 0

X: The classical style data on the natural course for skier 1 is not available.

**Table 6:** Number of cycles of the classical (DS, P-Off, KDP, DP) and skating (V2, V2A, V1, FS) techniques performed by skier 4 in the test set-1 and test set-2.

Test Set-1 Flat Course								Test Set-2 Natural Course			
Classical Style				Skating Style				Skating Style			
DS	P-Off	KDP	DP	V2	V2A	V1	FS	V2	V2A	V1	FS
649	511	378	569	366	447	631	480	72	13	219	0

**Table 7:** Five combinations of the sensors for which the CNN-LSTM based deep learning model is trained.

Sensor Configuration	Number of Sensors	Locations of Sensors
1	All 17 sensors (Whole-body)	Pelvis, chest, head, right and left shoulders, right and left upper arms, right and left forearms, right and left hands, right and left upper legs, right and left lower legs, right and left feet
2	11 sensors (Upper body only)	Pelvis, chest, head, right and left shoulders, right and left upper arms, right and left forearms, right and left hands
3	7 sensors (Lower body only)	Pelvis, right and left upper legs, right and left lower legs, right and left feet
4	5 sensors (Sports biomechanics configuration)	Pelvis, right and left hands, right and left feet
5	1 sensor (Pelvis only)	Pelvis

**Table 8:** Training dataset accuracies for the five different sensor configurations.

Sensor Configuration	17 Sensors (Whole Body)	11 Sensors (Upper Body)	7 Sensors (Lower Body)	5 Sensors (Sports Biomech.)	1 Sensor (Pelvis)
Accuracy	99.39%	97.96%	98.03%	97.82%	79.4%

**Table 9:** Confusion matrix for the training dataset for the sports biomechanics configuration of sensors.

	Predicted	DS	P-Off	KDP	DP	V2	V2A	V1	FS
True									
DS		339	0	0	0	0	0	0	0
P-Off		0	242	0	28	0	0	0	0
KDP		0	0	265	0	0	0	0	0
DP		0	9	1	264	0	0	0	0
V2		0	1	0	1	168	0	1	0
V2A		0	0	0	0	1	218	0	0
V1		0	0	0	0	3	0	305	0
FS		0	0	0	0	0	0	0	221

**Table 10:** Classification accuracies for the validation dataset for five different sensor configurations.

Sensor Configuration		Course & Technique	Natural Course		Flat Course		Mean Accuracy
	Skier		Classical Style	Skating Style	Classical Style	Skating Style	
Whole body sensors (17)	Skier 1		X	89.58%	78.82%	76.52%	81.64%
	Skier 2		97.31%	97.68%	89.15%	79.03%	90.79%
	Skier 3		93.67%	82.21%	91.06%	89.25%	89.04%
	Mean Accuracy		95.49%	89.82%	86.34%	81.60%	~87.00%
Upper body sensors (11)	Skier 1		X	87.85%	74.12%	71.97%	77.98%
	Skier 2		95.38%	95.78%	85.27%	72.58%	87.25%
	Skier 3		66.24%	82.21%	73.17%	88.17%	77.45%
	Mean Accuracy		80.81%	88.61%	77.52%	77.57%	~80.00%
Lower body sensors (7)	Skier 1		X	54.86%	78.82%	52.27%	61.98%
	Skier 2		92.31%	25.34%	85.27%	69.35%	68.07%
	Skier 3		85.23%	66.87%	90.24%	80.65%	80.75%
	Mean Accuracy		88.77%	49.02%	84.78%	67.42%	~70.00%
Sports biomechanics configuration (5)	Skier 1		X	76.04%	88.23%	71.96%	78.74%
	Skier 2		94.61%	96.87%	91.47%	82.26%	91.30%
	Skier 3		96.62%	82.21%	88.62%	92.47%	89.98%
	Mean Accuracy		95.61%	85.04%	89.44%	82.23%	~87.00%
Pelvis sensor only (1)	Skier 1		X	74.20%	80.21%	59.30%	71.24%
	Skier 2		56.37%	78.79%	74.44%	52.36%	64.81%
	Skier 3		59.32%	64.73%	68.80%	31.23%	56.02%
	Mean Accuracy		57.85%	72.57%	74.48%	47.63%	~64.00%

X: The classical style data on the natural course for skier 1 is not available.

**Table 11:** Confusion matrix for the natural course, classical style validation set of skier 3 when using the sports biomechanics configuration of sensors.

	Predicted	DS	P-Off	KDP	DP	V2	V2A	V1	FS
True									
DS		145	0	0	0	0	0	0	0
P-Off		0	1	0	1	0	0	0	0
KDP		0	0	4	0	1	0	0	0
DP		1	1	0	79	1	3	0	0
V2		0	0	0	0	0	0	0	0
V2A		0	0	0	0	0	0	0	0
V1		0	0	0	0	0	0	0	0
FS		0	0	0	0	0	0	0	0

**Table 12:** Confusion matrix for the natural course, skating style validation set of skier 3 when using the sports biomechanics configuration of sensors.

	Predicted	DS	P-Off	KDP	DP	V2	V2A	V1	FS
True									
DS		0	0	0	0	0	0	0	0
P-Off		0	0	0	0	0	0	0	0
KDP		0	0	0	0	0	0	0	0
DP		0	0	0	0	0	0	0	0
V2		0	0	0	0	86	3	0	0
V2A		0	0	0	0	1	5	23	1
V1		0	0	0	0	1	0	43	0
FS		0	0	0	0	0	0	0	0

**Table 13:** Confusion matrix for the flat course, classical style validation set of skier 3 when using the sports biomechanics configuration of sensors.

	Predicted	DS	P-Off	KDP	DP	V2	V2A	V1	FS
True									
DS		16	0	0	0	0	0	0	0
P-Off		0	22	0	5	0	0	0	0
KDP		0	6	27	1	0	0	0	0
DP		0	0	1	44	1	0	0	0
V2		0	0	0	0	0	0	0	0
V2A		0	0	0	0	0	0	0	0
V1		0	0	0	0	0	0	0	0
FS		0	0	0	0	0	0	0	0

**Table 14:** Confusion matrix for the flat course, skating style validation set of skier 3 when using the sports biomechanics configuration of sensors.

	Predicted	DS	P-Off	KDP	DP	V2	V2A	V1	FS
True									
DS		0	0	0	0	0	0	0	0
P-Off		0	0	0	0	0	0	0	0
KDP		0	0	0	0	0	0	0	0
DP		0	0	0	0	0	0	0	0
V2		0	0	1	0	20	1	1	0
V2A		0	0	0	1	0	30	0	1
V1		0	0	0	0	1	0	17	1
FS		0	0	0	0	0	0	0	19

**Table 15:** Classification accuracies for the first three skiers using the proposed deep learning model when a leave-one-out type of testing is performed using the sports biomechanics configuration of sensors.

	Course & Technique	Natural Course		Flat Course		Mean Accuracy
Skier		Classical Style	Skating Style	Classical Style	Skating Style	
Skier 1		X	90.35%	76.48%	74.50%	80.44%
Skier 2		76.20%	97.70%	68.65%	85.68%	82.06%
Skier 3		90.28%	78.50%	79.70%	55.00%	75.87%
Mean Accuracy		83.24%	88.85%	74.94%	71.73%	79.69%

X: The classical style data on the natural course for skier 1 is not available.

**Table 16:** Classification accuracies for the first three skiers using the k-nearest neighbors machine learning model when a leave-one-out type of testing is performed using the sports biomechanics configuration of sensors.

	Course & Technique	Natural Course		Flat Course		Mean Accuracy
Skier		Classical Style	Skating Style	Classical Style	Skating Style	
Skier 1		X	86.05%	57.25%	58.33%	67.21%
Skier 2		75.09%	87.76%	50.38%	53.33%	66.64%
Skier 3		83.71%	56.98%	57.25%	37.86%	58.95%
Mean Accuracy		79.40%	76.93%	54.96%	49.84%	65.28%

X: The classical style data on the natural course for skier 1 is not available.

**Table 17:** Classification accuracies for test set-1 and test set-2 for the five different sensor configurations.

Sensor Configuration	17 Sensors (Whole Body)	11 Sensors (Upper Body)	7 Sensors (Lower Body)	5 Sensors (Sports Biomech.)	1 Sensor (Pelvis)
**Test Set-1**	84.21%	84.80%	84.35%	87.20%	58.54%
**Test Set-2**	92.18%	84.83%	64.58%	95.10%	29.65%
**Mean Accuracy**	88.19%	84.82%	74.47%	91.15%	44.10%

**Table 18:** Confusion matrix for the test set-1, in which the test subject performs one of the techniques of one of the XC-skiing styles repeatedly on a flat course at a time for the sports biomechanics configuration of sensors.

	Predicted	DS	P-Off	KDP	DP	V2	V2A	V1	FS
True									
DS		310	0	0	0	0	0	0	0
P-Off		0	63	1	172	5	0	0	0
KDP		0	0	113	0	0	0	0	0
DP		0	74	0	221	0	0	0	0
V2		0	1	0	0	193	0	1	0
V2A		0	0	0	1	4	221	2	0
V1		0	0	0	0	1	3	319	0
FS		0	0	0	0	0	0	0	259

**Table 19:** Confusion matrix for test set-2 in which the test subject performs all skating techniques on a natural course simultaneously for the sports biomechanics configuration of sensors.

	Predicted	DS	P-Off	KDP	DP	V2	V2A	V1	FS
True									
DS		0	0	0	0	0	0	0	0
P-Off		0	0	0	0	0	0	0	0
KDP		0	0	0	0	0	0	0	0
DP		0	0	0	0	0	0	0	0
V2		0	0	0	1	66	2	3	0
V2A		0	0	0	0	0	13	0	0
V1		0	0	0	0	6	1	210	2
FS		0	0	0	0	0	0	0	2

**Table 20:** Classification accuracies for the validation dataset for five different sensor configurations using the k-nearest neighbors algorithm.

Sensor Configuration		Course & Technique	Natural Course		Flat Course		Mean Accuracy
	Skier		Classical Style	Skating Style	Classical Style	Skating Style	
Whole body sensors (17)	Skier 1		X	60.54%	61.59%	60.54%	60.89%
	Skier 2		91.70%	97.50%	83.67%	80.00%	88.22%
	Skier 3		88.26%	83.72%	80.15%	83.50%	83.91%
	Mean Accuracy		89.98%	80.59%	75.14%	74.68%	80.10%
Upper body sensors (11)	Skier 1		X	66.33%	47.83%	66.32%	60.16%
	Skier 2		84.43%	93.95%	77.56%	88.00%	85.99%
	Skier 3		83.34%	81.40%	71.76%	81.55%	79.51%
	Mean Accuracy		83.89%	80.56%	65.72%	78.62%	77.20%
Lower body sensors (7)	Skier 1		X	68.37%	58.70%	68.37%	65.15%
	Skier 2		82.70%	38.55%	77.56%	64.00%	65.70%
	Skier 3		85.98%	53.49%	81.68%	61.17%	70.58%
	Mean Accuracy		84.34%	53.47%	72.65%	64.51%	68.74%
Sports biomechanics configuration (5)	Skier 1		X	50.68%	52.17%	50.68%	51.18%
	Skier 2		86.85%	95.92%	83.67%	81.34%	86.95%
	Skier 3		82.58%	76.74%	81.00%	83.50%	80.96%
	Mean Accuracy		84.72%	74.45%	72.28%	71.84%	75.82%
Pelvis sensor only (1)	Skier 1		X	2.72%	25.36%	2.08%	10.05%
	Skier 2		31.83%	31.18%	15.74%	10.67%	22.36%
	Skier 3		34.09%	7.56%	18.32%	13.59%	18.39%
	Mean Accuracy		32.96%	13.82%	19.81%	8.78%	18.84%

X: The classical style data on the natural course for skier 1 is not available.

**Table 21:** Classification accuracies for test set-1 and test set-2 for the five different sensor configurations using the k-nearest neighbors algorithm.

Sensor Configuration	17 Sensors (Whole Body)	11 Sensors (Upper Body)	7 Sensors (Lower Body)	5 Sensors (Sports Biomech.)	1 Sensor (Pelvis)
**Test Set-1**	48.06%	33.58%	65.54%	68.82%	38.13%
**Test Set-2**	86.82%	71.96%	71.28%	78.04%	7.43%

**Table 22:** Comparison between the machine learning (ML-KNN) and deep learning (DL) methods on classification accuracies for test set-1 and test set-2 for the five different sensor configurations.

Sensor Configuration	17 Sensors (Whole Body)		11 Sensors (Upper Body)		7 Sensors (Lower Body)		5 Sensors (Sports Biomech.)		1 Sensor (Pelvis Body)	
Modeling method	ML-KNN	DL	ML-KNN	DL	ML-KNN	DL	ML-KNN	DL	ML-KNN	DL
Test set-1	48.1%	84.2%	33.6%	84.8%	65.5%	84.4%	68.8%	87.2%	38.1%	58.5%
Test set-2	86.8%	92.2%	72.0%	84.8%	71.3%	64.6%	78.0%	95.1%	7.4%	29.7%

## Appendix A

### Appendix A.1

Table A1, Table A2, Table A3 and Table A4 show the confusion matrices for the test set of skier 2 when using the sports biomechanics configuration of sensors.

**Table A1:** Confusion matrix for the natural course, classical style validation set of skier 2 when using the sports biomechanics configuration of sensors.

	Predicted	DS	P-Off	KDP	DP	V2	V2A	V1	FS
True									
DS		190	0	0	0	0	0	0	0
P-Off		0	0	0	0	0	0	0	0
KDP		0	0	7	0	0	0	0	0
DP		0	2	9	49	1	2	0	0
V2		0	0	0	0	0	0	0	0
V2A		0	0	0	0	0	0	0	0
V1		0	0	0	0	0	0	0	0
FS		0	0	0	0	0	0	0	0

**Table A2:** Confusion matrix for the natural course, skating style validation set of skier 2 when using the sports biomechanics configuration of sensors.

	Predicted	DS	P-Off	KDP	DP	V2	V2A	V1	FS
True									
DS		0	0	0	0	0	0	0	0
P-Off		0	0	0	0	0	0	0	0
KDP		0	0	0	0	0	0	0	0
DP		0	0	0	0	0	0	0	0
V2		0	0	0	2	93	3	0	0
V2A		0	1	0	8	0	25	0	0
V1		0	1	1	0	4	0	593	3
FS		0	0	0	0	0	0	0	0

**Table A3:** Confusion matrix for the flat course, classical style validation set of skier 2 when using the sports biomechanics configuration of sensors.

	Predicted	DS	P-Off	KDP	DP	V2	V2A	V1	FS
True									
DS		48	1	0	0	0	0	0	0
P-Off		3	50	1	0	0	0	0	0
KDP		2	3	20	1	0	0	0	0
DP		0	0	0	0	0	0	0	0
V2		0	0	0	0	0	0	0	0
V2A		0	0	0	0	0	0	0	0
V1		0	0	0	0	0	0	0	0
FS		0	0	0	0	0	0	0	0

**Table A4:** Confusion matrix for the flat course, skating style validation set of skier 2 when using the sports biomechanics configuration of sensors.

	Predicted	DS	P-Off	KDP	DP	V2	V2A	V1	FS
True									
DS		0	0	0	0	0	0	0	0
P-Off		0	0	0	0	0	0	0	0
KDP		0	0	0	0	0	0	0	0
DP		0	0	0	0	0	0	0	0
V2		0	0	0	0	10	2	1	0
V2A		0	0	0	1	2	20	0	1
V1		0	0	1	0	2	0	12	0
FS		0	0	0	0	0	1	0	9

### Appendix A.2

Table A5, Table A6 and Table A7 show the confusion matrices for the test set of skier 1 when using the sports biomechanics configuration of sensors.

**Table A5:** Confusion matrix for the natural course, skating style validation set of skier 1 when using the sports biomechanics configuration of sensors.

	Predicted	DS	P-Off	KDP	DP	V2	V2A	V1	FS
True									
DS		0	0	0	0	0	0	0	0
P-Off		0	0	0	0	0	0	0	0
KDP		0	0	0	0	0	0	0	0
DP		0	0	0	0	0	0	0	0
V2		0	0	0	2	34	15	8	1
V2A		0	0	0	0	2	27	2	0
V1		0	0	0	0	5	30	158	4
FS		0	0	0	0	0	0	0	0

**Table A6:** Confusion matrix for the flat course, classical style validation set of skier 1 when using the sports biomechanics configuration of sensors.

	Predicted	DS	P-Off	KDP	DP	V2	V2A	V1	FS
True									
DS		30	1	2	0	0	0	0	0
P-Off		0	10	1	2	0	0	0	0
KDP		1	3	35	0	0	0	0	0
DP		0	0	0	0	0	0	0	0
V2		0	0	0	0	0	0	0	0
V2A		0	0	0	0	0	0	0	0
V1		0	0	0	0	0	0	0	0
FS		0	0	0	0	0	0	0	0

**Table A7:** Confusion matrix for the flat course, skating style validation set of skier 1 when using the sports biomechanics configuration of sensors.

	Predicted	DS	P-Off	KDP	DP	V2	V2A	V1	FS
True									
DS		0	0	0	0	0	0	0	0
P-Off		0	0	0	0	0	0	0	0
KDP		0	0	0	0	0	0	0	0
DP		0	0	0	0	0	0	0	0
V2		0	0	0	0	19	21	4	0
V2A		0	0	0	0	3	54	0	3
V1		0	0	0	0	0	0	7	0
FS		0	0	0	1	0	2	3	15

### Appendix A.3

**Table A8:** Classification accuracies for the first three skiers using the proposed deep learning model when a leave-one-out type of testing is performed using four different sensor configurations of sensors.

Sensor Configuration		Course & Technique	Natural Course		Flat Course		Mean Accuracy	
	Skier		Classical Style	Skating Style	Classical Style	Skating Style		
Whole body sensors (17)	Skier 1		X	89.4%	82.3%	76.5%	82.7%
	Skier 2		97.7%	96.2%	84.5%	88.7%	91.8%
	Skier 3		92.4%	61.4%	82.9%	54.8%	72.9%
	Mean Accuracy		95.0%	82.3%	83.2%	73.3%	~83.0%
Upper body sensors (11)	Skier 1		X	82.4%	67.6%	74.2%	74.7%
	Skier 2		73.5%	68.8%	76.5%	64.1%	70.7%
	Skier 3		66.2%	42.3%	78.5%	44.1%	57.8%
	Mean Accuracy		69.9%	64.5%	74.2%	60.8%	~68%
Lower body sensors (7)	Skier 1		X	24.2%	74.9%	66.3%	55.1%
	Skier 2		94.6%	24.2%	86.8%	67.7%	68.3%
	Skier 3		91.1%	68.7%	86.2%	50.5%	74.1%
	Mean Accuracy		92.9%	39.0%	82.6%	61.5%	~66%
Pelvis sensor only (1)	Skier 1		X	24.0%	77.6%	56.1%	52.6%
	Skier 2		64.6%	85.3%	51.1%	38.7%	59.9%
	Skier 3		62.4%	20.2%	82.1%	21.5%	46.5%
	Mean Accuracy		63.5%	43.1%	70.3%	38.8%	~53%

X: The classical style data on the natural course for skier 1 is not available.

**Table A9:** Classification accuracies for the first three skiers using the k-nearest neighbors algorithm when a leave-one-out type of testing is performed using four different sensor configurations of sensors.

Sensor Configuration		Course & Technique	Natural Course		Flat Course		Mean Accuracy
	Skier		Classical Style	Skating Style	Classical Style	Skating Style	
Whole body sensors (17)	Skier 1		X	89.1%	65.2%	65.4%	73.2%
	Skier 2		69.5%	82.6%	60.8%	78.7%	72.9%
	Skier 3		81.4%	74.4%	70.2%	56.3%	70.6%
	Mean Accuracy		75.5%	82.0%	65.4%	66.8%	~72%
Upper body sensors (11)	Skier 1		X	50.3%	46.4%	61.1%	52.6%
	Skier 2		66.8%	51.7%	61.3%	58.7%	59.6%
	Skier 3		64.4%	73.3%	49.6%	55.3%	60.7%
	Mean Accuracy		65.6%	58.4%	52.4%	58.4%	~59%
Lower body sensors (7)	Skier 1		X	81.3%	56.5%	50.7%	62.8%
	Skier 2		49.8%	51.7%	48.8%	38.7%	47.3%
	Skier 3		81.1%	38.9%	62.6%	36.9%	54.9%
	Mean Accuracy		65.4%	57.3%	56.0%	42.1%	~55%
Pelvis sensor only (1)	Skier 1		X	2.4%	30.4%	2.1%	11.7%
	Skier 2		21.8%	10.9%	15.7%	9.3%	14.5%
	Skier 3		40.1%	2.9%	24.4%	7.8%	18.8%
	Mean Accuracy		30.9%	5.4%	23.5%	6.4%	~15%

X: The classical style data on the natural course for skier 1 is not available.
